# Supplementary material for: Baseline Characterization of the Gut Microbiota of Field and Colony Populations of Phlebotomus tobbi and Preliminary Assessment of the Anti-Leishmanial Activity of Cultivable Bacteria
Source: Pathogens. 2026 Jun 23;15(7):658. doi: 10.3390/pathogens15070658 (PMC13416292; doi:10.3390/pathogens15070658)
Supplement: Supplementary file 1 [file pathogens-15-00658-s001.zip › pathogens-4357168-File S1/Taxa2ASV_Decomposer/Genus/g__Pantoea/heatmap_without_clustering.pdf]

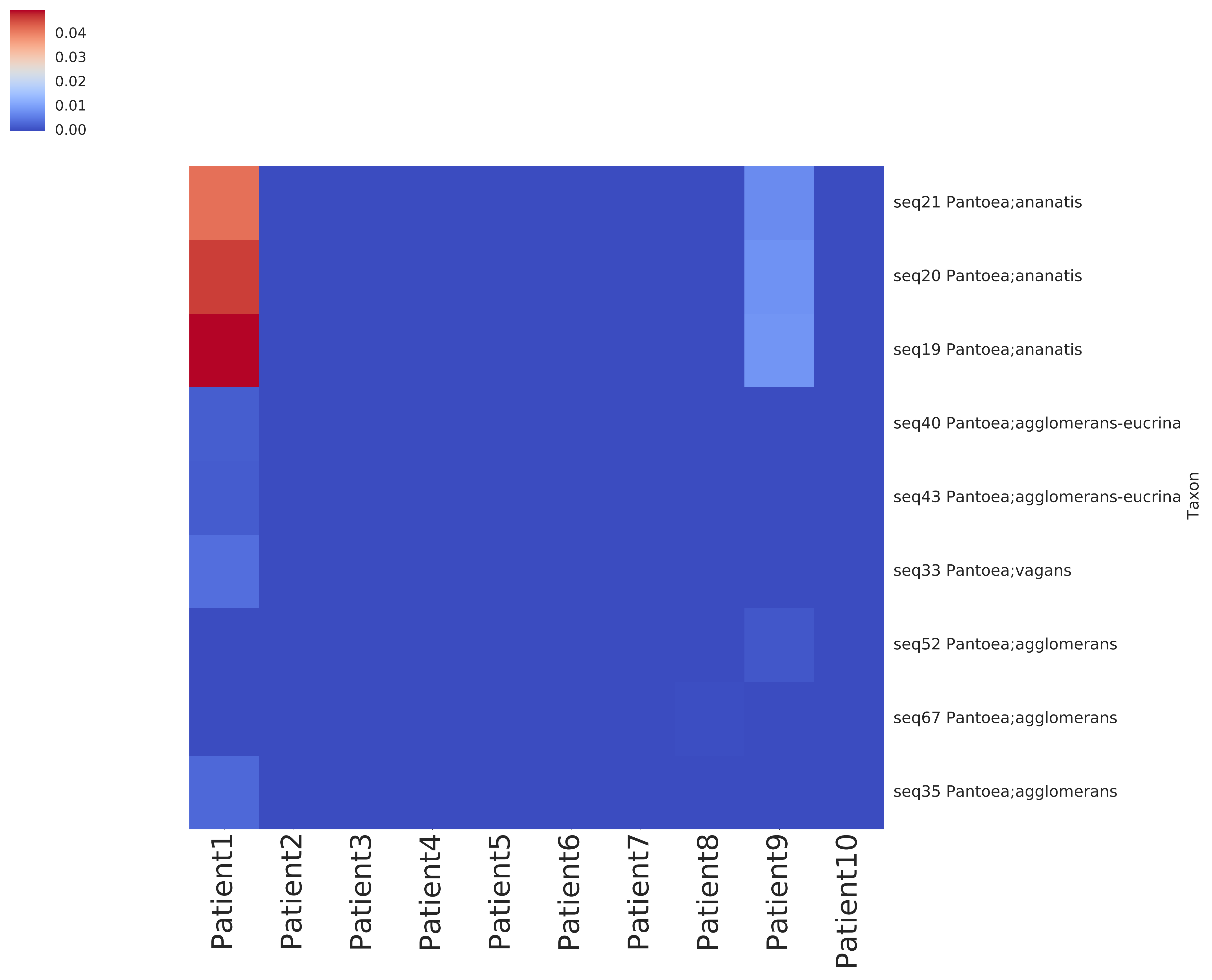
0.04
0.03
0.02
0.01
0.00
seq21 Pantoea;ananatis
seq20 Pantoea;ananatis
seq19 Pantoea;ananatis
seq40 Pantoea;agglomerans-eucrina
seq43 Pantoea;agglomerans-eucrina
seq33 Pantoea;vagans
seq52 Pantoea;agglomerans
seq67 Pantoea;agglomerans
seq35 Pantoea;agglomerans
Taxon
Patient1
Patient2
Patient3
Patient4
Patient5
Patient6
Patient7
Patient8
Patient9
Patient10
